# Phenotype, function, and differentiation potential of human monocyte subsets

**DOI:** 10.1371/journal.pone.0176460

**Published:** 2017-04-26

**Authors:** Lisa B. Boyette, Camila Macedo, Kevin Hadi, Beth D. Elinoff, John T. Walters, Bala Ramaswami, Geetha Chalasani, Juan M. Taboas, Fadi G. Lakkis, Diana M. Metes

**Affiliations:** 1 Thomas E. Starzl Transplantation Institute, Department of Surgery, University of Pittsburgh School of Medicine, Pittsburgh, PA, United States; 2 Department of Medicine, University of Pittsburgh School of Medicine, Pittsburgh, PA, United States; 3 Department of Oral Biology, University of Pittsburgh School of Dental Medicine, Pittsburgh, PA, United States; 4 Department of Bioengineering, University of Pittsburgh Swanson School of Engineering, Pittsburgh, PA, United States; 5 McGowan Institute for Regenerative Medicine, University of Pittsburgh School of Medicine, Pittsburgh, PA, United States; 6 Department of Immunology, University of Pittsburgh School of Medicine, Pittsburgh, PA, United States; Universitatsklinikum Freiburg, GERMANY

## Abstract

Human monocytes have been grouped into classical (CD14^++^CD16^−^), non-classical (CD14^dim^CD16^++^), and intermediate (CD14^++^CD16^+^) subsets. Documentation of normal function and variation in this complement of subtypes, particularly their differentiation potential to dendritic cells (DC) or macrophages, remains incomplete. We therefore phenotyped monocytes from peripheral blood of healthy subjects and performed functional studies on high-speed sorted subsets. Subset frequencies were found to be tightly controlled over time and across individuals. Subsets were distinct in their secretion of TNFα, IL-6, and IL-1β in response to TLR agonists, with classical monocytes being the most producers and non-classical monocytes the least. Monocytes, particularly those of the non-classical subtype, secreted interferon-α (IFN-α) in response to intracellular TLR3 stimulation. After incubation with IL-4 and GM-CSF, classical monocytes acquired monocyte-derived DC (mo-DC) markers and morphology and stimulated allogeneic T cell proliferation in MLR; intermediate and non-classical monocytes did not. After incubation with IL-3 and Flt3 ligand, no subset differentiated to plasmacytoid DC. After incubation with GM-CSF (M1 induction) or macrophage colony-stimulating factor (M-CSF) (M2 induction), all subsets acquired macrophage morphology, secreted macrophage-associated cytokines, and displayed enhanced phagocytosis. From these studies we conclude that classical monocytes are the principal source of mo-DCs, but all subsets can differentiate to macrophages. We also found that monocytes, in particular the non-classical subset, represent an alternate source of type I IFN secretion in response to virus-associated TLR agonists.

## Introduction

Monocytes play an important role in immune defense, inflammation, and homeostasis by sensing their local environment, clearing pathogens and dead cells, and initiating adaptive immunity, as well as providing a progenitor pool that contributes to inflammatory DCs and replenishes some tissue macrophages [1–4]. Monocytes also contribute to tissue repair, [1–7] further underscoring their relevance to both health and disease. Similar to other cell types with primary and progenitor functions, monocytes are a heterogeneous and plastic cell population, with context-dependent functions attributed to different subsets [8,9]. This has generated controversy in the literature, where functions of a specific monocyte subset responding to a specific stimulus, for example infection, in a specific site *in vivo* [10–15] have been extrapolated to that subset under all conditions, which is frequently inaccurate [16]. Therefore, a clearer definition of monocyte subset function is needed.

Monocyte subset function has been more extensively studied in mouse models than in humans. In the mouse Ly6C^+/high^ (Gr1^+/high^) or inflammatory monocytes circulate in the blood and egress into tissues following infection, at which point they differentiate into macrophages and DCs, producing inflammatory cytokines and reactive oxygen species, stimulating effector T-cell proliferation, and mediating tissue repair. These cells also contribute to a population of monocyte-derived suppressor cells that inhibit T-cell function in cancer and autoimmune models. Ly6C^−/low^ (Gr1^−/low^) monocytes adopt a patrolling phenotype, characterized by long-range crawling along the luminal surface of small vessels, that allows them to survey the organism for tissue damage in the form of dying and infected cells and mediate their disposal [17–19].

Human monocyte subpopulations that correspond in phenotype and function to those present in mice are the CD14^++^CD16^−^ (classical), which account for 80–90% of peripheral blood monocytes, and the CD14^dim^CD16^++^ (non-classical) subpopulations. The former corresponds to the Ly6C^+/high^ inflammatory subset while the latter corresponds to Ly6C^-/low^ patrolling subset in mice. Microarray-based gene expression profiling has confirmed that differential gene expression profiles observed in mouse monocyte subsets are conserved in human monocyte subpopulations [3,20–24]. However, it should be noted that many *in vivo* observations of patrolling monocyte function in mouse models take place during a stage when those cells are tethered to the vasculature, which may reflect a more committed state than that of non-classical human monocytes obtained via a peripheral blood draw. An intermediate CD14^++^CD16^+^ subset has also been identified in human blood, and the functions previously attributed to all CD16^+^ monocytes appear to be distinct for intermediate versus non-classical (CD14^dim^) subsets [21]. Functional observations of human monocyte subsets documented in the literature are somewhat controversial, with different responses observed depending on the context of their activation. Microarray and functional studies of human and other primate monocyte subsets in one case showed a closer association of intermediate monocytes with classical monocytes [21] and in others with non-classical monocytes [25,26]. Although there is agreement that functionally significant monocyte heterogeneity is conserved among mammals [27], further study of these subsets and their capabilities is clearly indicated.

It is likely that these diverse functions attributed generally to monocytes are due to the presence of multiple monocyte subpopulations responding to activation in a variety of contexts over time [3,6,28]. A thorough understanding of the homeostasis, function, and differentiation potential of human monocyte subsets from healthy subjects is therefore a pre-requisite for establishing their roles in human disease. Such understanding should enable more specific targeting of innate responses in the setting of infection, inflammation, autoimmunity, cancer, and transplantation as well as the development of monocyte-based biomarkers that measure either disease activity or response to therapy in these conditions [29]. In the current study, we aimed at establishing a baseline understanding of how each subset of the monocyte compartment in healthy adult humans behaves under a limited set of clearly defined, well controlled, commonly used *ex vivo* laboratory conditions in order to uncover key differences between the three human monocyte subsets related to their hardwired function and differentiation potential.

## Materials and methods

### Immunophenotyping

Peripheral blood was drawn from consenting healthy adult subjects with IRB approval at the University of Pittsburgh (IRB#00608014). Cell counts were obtained using a Coulter Counter Analyzer (Beckman Coulter). Monocytes were immunophenotyped in 200 μL of fresh whole blood. Non-specific binding was blocked using 10% mouse serum (Invitrogen). Cells were stained for 30 minutes with monoclonal antibodies (mAbs) against the markers described below, treated with Fix/Lyse buffer (BD Biosciences) according to the manufacturer’s protocol, and washed twice in FACS buffer (PBS supplemented with 1% FCS and 0.05% sodium azide) prior to analysis.

Lymphoid cells were excluded using fluorescently preconjugated primary antibodies against lineage markers CD3, CD4, CD15, CD19, CD56, and NKp46 after exclusion of doublets and HLA-DR^−^ cells (all from BD). Monocyte subsets were identified according to their expression of CD14 and CD16, and percent of total monocytes and absolute number was calculated for each subset. A panel of markers was interrogated for each sample using fluorescently preconjugated primary antibodies against CD11b, CD11c, CD62L, CD163, CD80, CD47, SIRPα, PD-1, PD-L1, PD-L2 (BD) and CX3CR1 (eBioscience). Marker expression was measured using a BD LSR Fortessa flow cytometer and data were analyzed using FlowJo (Tree Star). Isotype controls and/or FMO controls for each marker were used to calculate delta mean fluorescence intensity for the population.

### Isolation of human monocyte subsets

Leukocyte concentrates were purchased from the Central Blood Bank of Pittsburgh. Red blood cells (RBC) were lysed using an ammonium chloride buffer (Stem Cell Technologies) according to the manufacturer’s protocol. PBMCs were rinsed in wash buffer twice, then incubated at 37°C in FCS containing 5mM EDTA to remove any remaining platelets [30,31]. After washing, monocytes were negatively selected using an EasySep Human Monocyte Enrichment Kit without CD16 Depletion (Stem Cell Technologies) according to the manufacturer’s protocol. Remaining cells were stained for FACS with anti-CD14, -16, -HLA-DR, and lineage exclusion markers as described for phenotyping above. High-speed sorting was performed on a BD FACSAria to yield high-purity monocyte subset populations (>95%). Cells were cultured in complete medium consisting of RPMI-1640 (Cellgro) supplemented with 2 mML-glutamine, 10mM HEPES, 100 IU/mL penicillin/streptomycin and 10% heat-inactivated fetal calf serum (FCS) (all from Gibco) except where other media formulations are indicated.

### Cell imaging

Sorted monocytes were imaged after being seeded in chamber slides (ibidi) and allowed to adhere. Cells were fixed in 4% paraformaldehyde, permeabilized with a saponin solution, and stained with Hoescht dye and fluorescently labeled phalloidin (both from Molecular Probes), and mAbs against β-tubulin (BD) and vinculin (Abcam). Cell morphology was captured on an Eclipse TiE epifluorescent microscope with NIS Elements deconvolution software (Nikon).

### TLR stimulation and luminex assays of cytokine production

Sorted monocyte subsets were cultured overnight (18 hours) in RPMI-1640 with each agonist from the Human Toll-like receptor (TLR) 1–9 kit according to protocols supplied by the manufacturer (InvivoGen), with use of the following working concentrations: pam3CSK4 (1μg/mL), HKLM (10^8^ cells/mL), poly(I:C) (10 μg/mL), LPS (1 μg/mL), flagellin (10 μg/mL), FSL-1 (1μg/mL), imiquimod (10 μg/mL), ssRNA40 (10 μg/mL), ODN2006 (5 μM). Inflammatory cytokines were quantified in culture supernatants by Milliplex Luminex bead-linked immunoassay according to the manufacturer’s protocol (EMD Millipore). Luminex beads were read and cytokine concentration calculated on a Bio-Plex Luminex reader (BioRad). All samples and standards were measured in duplicate and measurements were normalized to baseline reactivity of unstimulated cells for each subject and cytokine.

A similar method was used to determine concentrations of IFN-α following induction to plasmacytoid DC (pDC) differentiation and stimulation with the intracellular TLR agonists Poly(I:C), ssRNA40, and CpG ODN 2216 in the presence of the lipid-based transfection reagent LyoVec (all from InvivoGen); this method was also used to quantify macrophage-associated cytokines following induction to classical (M1) and alternative (M2) macrophage differentiation. Supernatants were collected after overnight incubation from undifferentiated monocyte cultures and treated cultures, cryopreserved at -80°C, then analyzed as described above.

### Monocyte differentiation

Monocyte subsets were induced to differentiate to one of four phenotypes following isolation: (1) mo-DCs by culturing in AIM-V medium supplemented with 10% heat-inactivated fetal calf serum (FCS) (Gibco), 1000 U/mL recombinant human (rh) granulocyte–macrophage colony-stimulating factor (GM-CSF) (Berlex) and 1000 U/mL rhIL-4 (R&D Systems) for seven days; (2) pDCs by culturing in complete medium supplemented with 20 ng/mL rhIL-3 and 100 ng/mL rhFlt3 ligand (both from Miltenyi Biotec) for seven days; (3) classical (M1) macrophages by culturing in complete medium supplemented with 50 ng/mL rhGM-CSF for five days; or (4) alternative (M2) macrophages by culturing in complete medium supplemented with 100 ng/mL rh macrophage colony-stimulating factor (M-CSF) (Miltenyi Biotec) for five days. Differentiation studies were performed on tissue culture-treated plastic (Sigma).

### Interrogation of surface markers on differentiated populations

Expression of surface markers of interest was measured by flow cytometry as described above following induction to terminal differentiation using fluorescently preconjugated primary mAbs against CD209, CD1a, CD40, CD86, CD54, CD80, CD83, HLA-DR,DP,DQ, CD71, CD206, CD123, Flt3 (all from BD), BDCA-2, (eBioscience), and EMR1 (Abcam), in addition to the other mAbs described above.

### CFSE-MLR

MLR were performed by incubating each subset of interest in a 1:5 ratio with carboxyfluorescein diacetate succinimidyl ester (CFSE)-dyed (Molecular Probes) allogeneic responder PBMCs for 5 days at 37°C in 5% CO_2_-air. Proliferation of allo-activated CD4^+^ or CD8^+^ T cells was identified after cell surface staining with mAbs against CD3 (BD), CD8 (BD), and with a dead cell stain kit (Molecular Probes) and by quantifying CFSE dilution. For IFN-γ (BD) intracellular staining, cultured cells were re-stimulated *in vitro* for 4 h with 4 μg/mL anti-CD3 (BD) and 2 μg/mL anti-CD28 (R&D) mAbs in the presence of Golgi-Plug (BD). Data acquisition and analysis was performed as described for immunophenotyping.

### Phagocytosis assays

Phagocytosis assays were performed by incubating monocytes with 1 μm fluorescent polystyrene beads (Spherotech) for 30 minutes at 37°C. Cells were rinsed twice in FACS buffer, and fluorescent signal was measured by flow cytometry.

### Statistical analyses

Differences in surface marker expression were detected using repeated measures one-way ANOVA with matched subjects, followed by Bonferroni post-testing to compare means of each group to one another using Prism. Luminex data were normalized for each cytokine with each subject serving as their own control and analyzed using repeated measures one-way ANOVA. All statistical analyses were performed using GraphPad Software.

## Results

### Monocyte subset frequencies are tightly regulated

**Fig 1:**
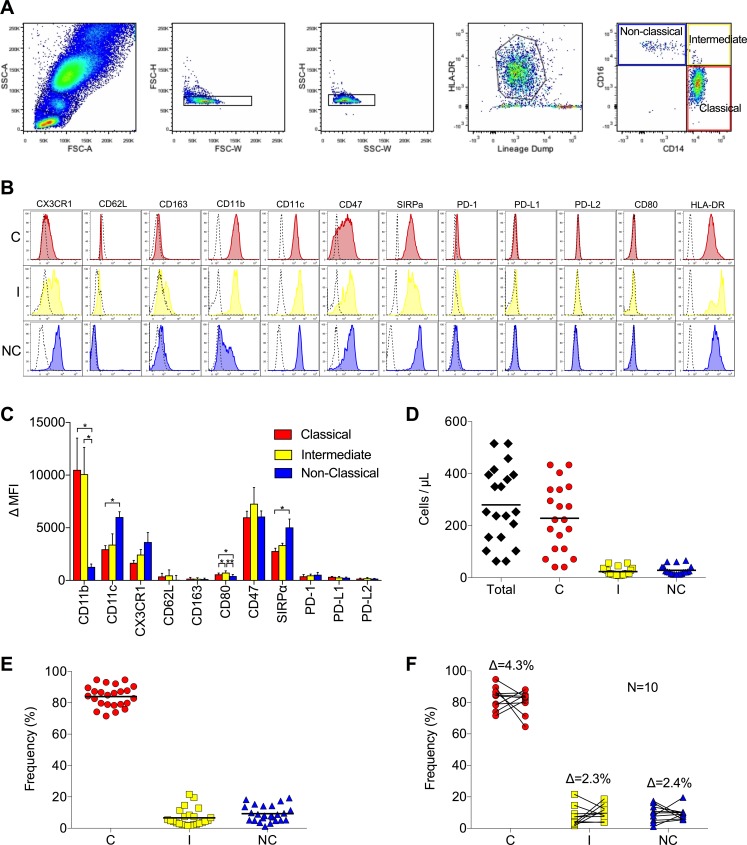
Monocyte phenotyping reveals tightly controlled distribution of subsets. (A) Gating strategy for phenotyping human monocyte subsets, including (from left to right) size discrimination, doublet exclusion, selection of HLA-DR^+^lin^−^ cells, and separation according to expression of CD14 and CD16. (B) Representative histograms of cell surface markers on monocyte subsets from one of 25 healthy subjects phenotyped (C = classical, I = intermediate, NC = non-classical). Classical monocytes (red), intermediate monocytes (yellow), and non-classical monocytes (blue) are shown overlaid on negative controls (dashed). (C) Mean expression (±SD) of surface markers on all subjects phenotyped. (D) Absolute numbers of total (black), classical (red), intermediate (yellow), and non-classical monocytes (blue) in circulation (bar = mean). (E) Frequency of each subset as a percentage of total circulating monocytes. (F) Change in the frequency of each subset in each of 10 subjects re-phenotyped 4–6 months following initial phenotyping. The average change in frequency (Δ) of each subset is shown.

Gating of human monocyte subsets according to size, granularity, HLA-DR expression, and expression of CD14 and CD16 (after exclusion of doublets and lineage^+^ cells) yielded three subsets: the classical subset (CD14^++^CD16^−^), the intermediate subset (CD14^++^CD16^+^), and the non-classical subset (CD14^dim^CD16^++^) (Fig 1A). Analysis of surface markers on cells from 25 healthy subjects revealed that these subsets are distinct from one another in their expression of CD11b (p = 0.0095), with higher expression on the classical and intermediate subsets, and CD11c (p = 0.026) and CX3CR1 (p = 0.15) (fractalkine receptor), with highest expression on the non-classical subset (Fig 1B and 1C). CD62L and CD163 were expressed at low levels on all subsets, with no detectable difference between subsets. Interrogation of co-stimulatory and co-inhibitory molecules revealed that all monocytes express low levels of CD80 (B7) at baseline, with intermediate monocytes consistently expressing slightly more. All subsets expressed the co-inhibitory molecule CD47 and its receptor SIRPα, with non-classical monocytes having the highest SIRPα expression (p = 0.028). The number of total monocytes in circulation varied widely across healthy individuals, ranging from 63.42/μL to 515.07/μL (Fig 1D). Monocyte subset frequencies were found to be tightly regulated across individuals, however, with the following means for each subset: 84% classical, 95% CI (81.1, 86.8), 6.7% intermediate (4.3, 9), and 9.3% non-classical (7.1, 11.4) (Fig 1E). Over time (4–6 months) the average change in subset frequencies for any given individual was not significant: 4.3% difference in classical frequency, 2.3% difference for intermediate, and 2.4% difference for non-classical (Fig 1F).

### Morphology of sorted monocyte subsets

**Fig 2:**
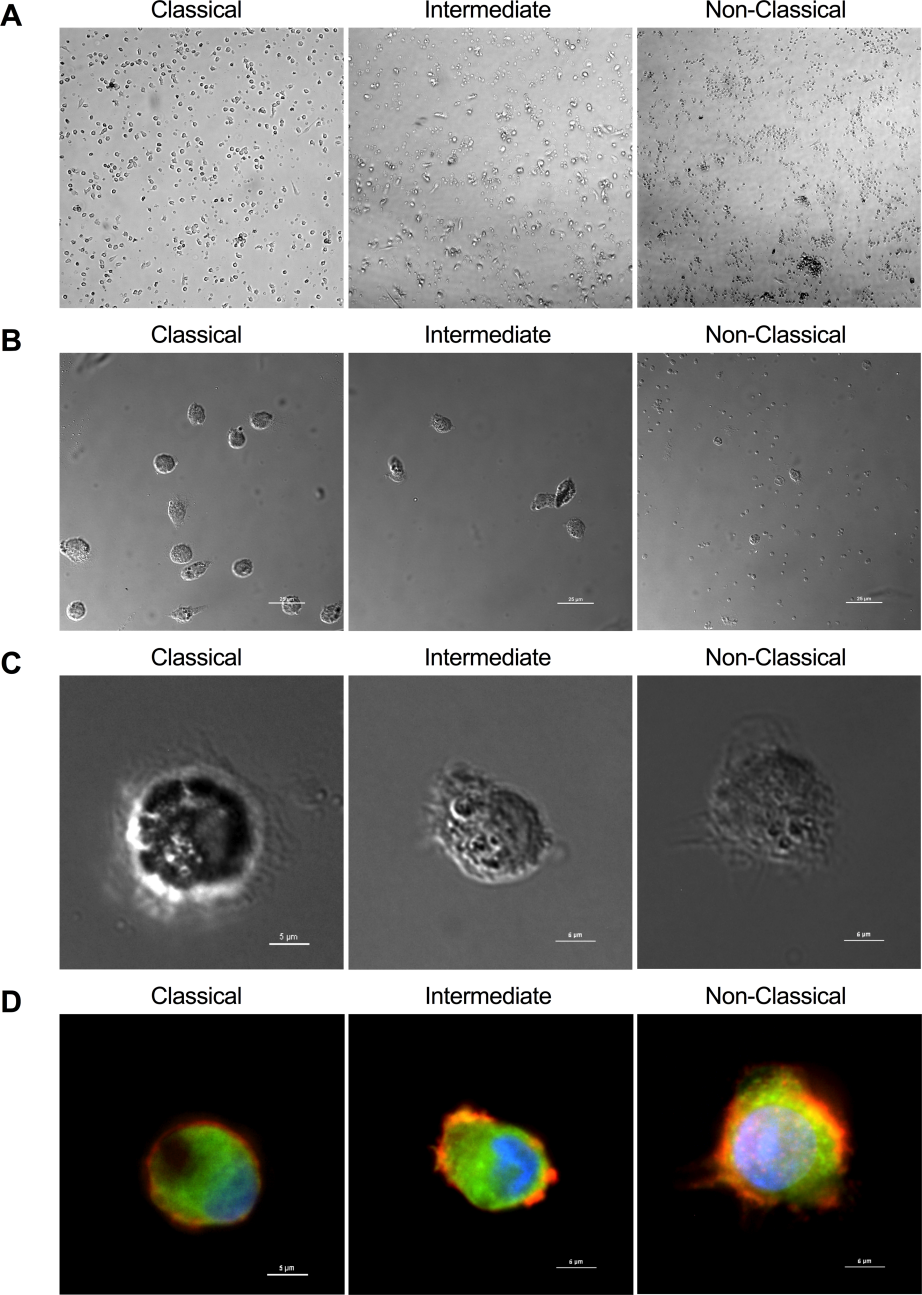
Morphology of sorted monocyte subsets. **(A)** Low power magnification of monocyte subsets after magnetic enrichment and high-speed sorting from leukocyte concentrates (photomicrographs taken at 10X). **(B)** Differential interference contrast (DIC) image of monocyte subsets after two-day culture and adherence to substrate at low magnification (upper panel) and high magnification (lower panel). **(C)** Fluorescent image of the cells shown in lower panel B, with actin (orange), vinculin (green), β-tubulin (green) and nucleus (blue) stained to show morphological features.

Immediately after magnetic enrichment and high-speed sorting from leukocyte concentrates, all monocyte subsets appeared as small, round, homogeneous cell populations by bright-field microscopy (Fig 2A). After two days of culture on plastic in complete medium without added growth factors, classical monocytes adhered well and appeared to proliferate (Fig 2B). Non-classical monocytes were smaller than the other subsets and began to die, with a few adhering and demonstrating early conversion to macrophage morphology, as evidenced by macrophage-specific morphological features, such as punctate actin-rich podosomes and vinculin-rich focal adhesions (Fig 2B and 2C). The intermediate subset displayed behavior in-between that of the classical and non-classical subsets (Fig 2B and 2C).

### Monocyte subsets are distinct in secretion of inflammatory cytokines

**Fig 3:**
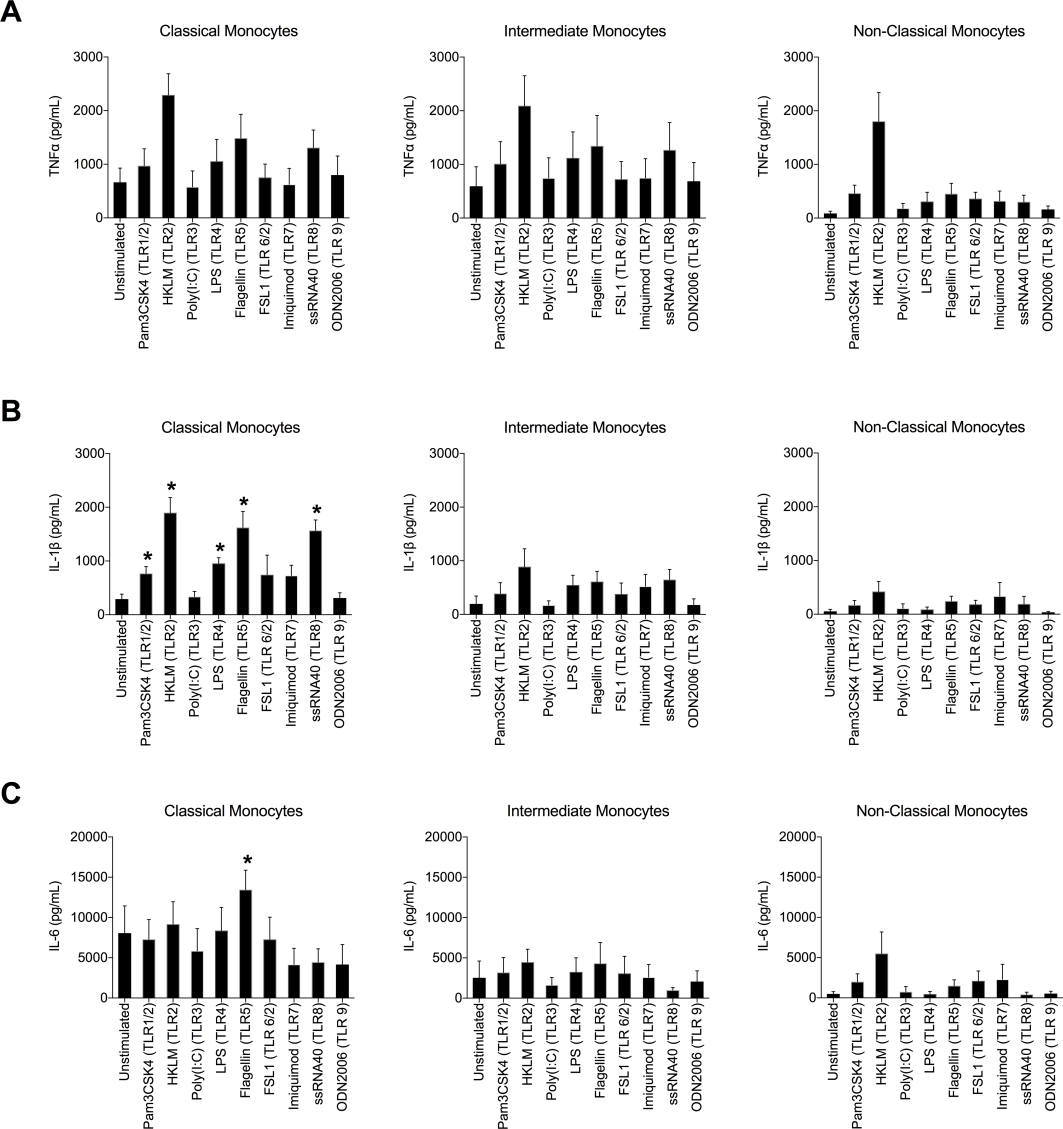
Cytokine secretion by sorted monocyte subsets stimulated with agonists to TLRs 1–9. Secretion of TNFα **(A)**, IL-1β **(B)** and IL-6 **(C)** by monocyte subsets (mean ± SD, n = 6) in response to extracellular TLR agonists pam3CSK4 (1μg/mL), HKLM (10^8^ cells/mL), poly(I:C) (10 μg/mL), LPS (1 μg/mL), flagellin (10 μg/mL), FSL-1 (1μg/mL), imiquimod (10 μg/mL), ssRNA40 (10 μg/mL), and ODN2006 (5 μM) in culture for 18 hours is shown (*p<0.05).

Freshly isolated classical, intermediate, and non-classical subsets were distinct in their secretion of IL-1β, IL-6, and TNFα in response to overnight stimulation with agonists to Toll-like receptors 1–9, with classical monocytes generally being the best producers and non-classical monocytes being poor producers (Fig 3). Intermediate monocytes produced levels of TNFα comparable to classical monocytes in response to all stimulants, which is consistent with previous reports (Fig 3A) [32]. Secretion of IL-1β in response to several TLR agonists was significantly higher in the classical subset than others (Fig 3B). Similarly, secretion of IL-6 by the classical subset was higher than the other subsets, particularly in response to Flagellin, a bacterial protein that signals through TLR5 (Fig 3C). We also measured levels of IL-10, IL-12p70, and IL-23, but we observed no significant secretion of these cytokines by any undifferentiated monocyte subset (data not shown).

### Classical monocytes are the primary source of monocyte-derived DC

**Fig 4:**
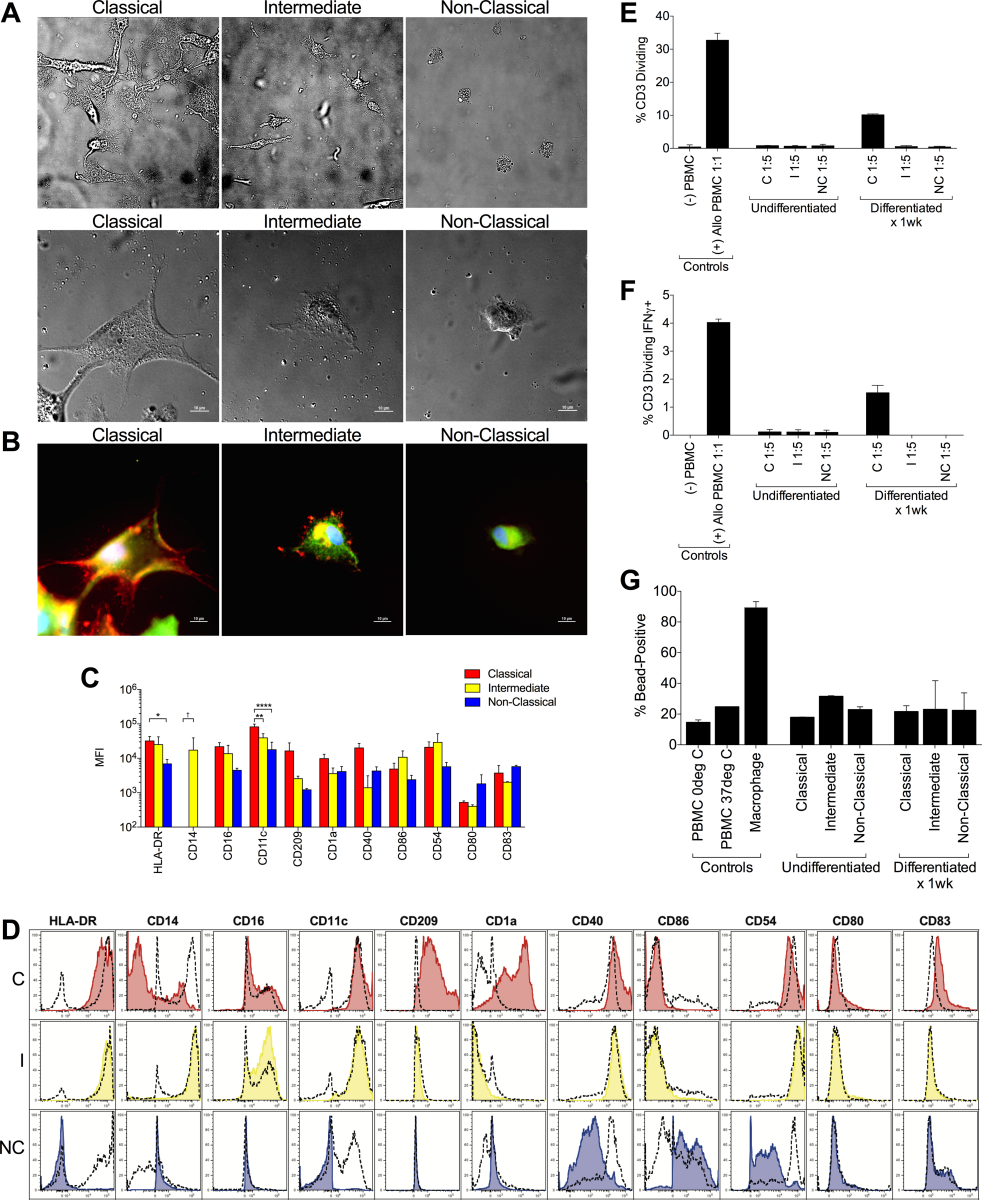
Classical monocytes differentiate to monocyte-derived DCs. **(A)** DIC images of sorted monocyte subsets after seven days in DC differentiation medium, with classical monocytes displaying fully differentiated DC morphology, intermediate monocytes also displaying DC morphology, although the cells are fewer and smaller, and non-classical monocytes showing no differentiation to DC in the few living cells, but otherwise evidence of cell death (upper panel, photomicrographs taken at 20X). DIC images (bottom panels) were captured at 60X (bar = 10μm). **(B)** Fluorescent images of the cells pictured in lower panel of **A**, with actin (orange), vinculin (green), β-tubulin (green) and nucleus (blue) stained to show morphological features (bar = 10μm). **(C)** MFI (mean ±SD) of surface marker expression for all subjects (n = 5) (* p<0.05, ** p<0.01, **** p<0.0001, † p = 0.06). **(D)** Representative histograms from one subject (C = classical, I = intermediate, NC = non-classical). Plots from each subset–classical monocytes (red), intermediate monocytes (yellow), and non-classical (blue)–cultured in DC differentiation medium for seven days are shown overlaid on plots from control cultures for each subset cultured in unsupplemented complete medium for seven days (dashed). **(E)** T cell proliferation in allogeneic MLRs using mo-DCs derived from classical monocytes (right bracket), undifferentiated unfractionated monocytes (center bracket), or PBMC (left bracket) as stimulators are shown (mean ± SD, 2 technical replicates from one subject). **(F)** IFN-γ production in dividing T cells in allogeneic MLRs shown in E. **(G)** Phagocytosis assay using DC differentiated from classical, intermediate, and nonclassical monocytes (right bracket) or undifferentiated monocytes (center bracket) Controls include PBMCs incubated with beads at 0°C and 37°C, as well as monocyte-derived macrophages (left bracket). Bars represent mean ± SD of 2 technical replicates from one subject.

We examined the potential of each subset to differentiate to various terminal fates in the presence or absence of differentiation cues (growth factors and cytokines). After culture in mo-DC induction medium (GM-CSF and IL-4), classical monocytes acquired mo-DC morphology, similar to what has been described for unfractionated monocytes [33]. As early as two days after induction with mo-DC medium, bright field images of sorted monocyte subsets showed that classical monocytes were already forming dendrites, with sparse macrophage-like cells intermixed; intermediate monocytes displayed a mix of highly adherent macrophage-like morphology and occasional cells with dendrites; and non-classical monocytes showed differentiation of a few cells to macrophage-like morphology, but massive cell death otherwise (data not shown). After one week of culture, classical monocytes exhibited fully differentiated DC characteristics, with large, adherent morphology and many dendrites (Fig 4A and 4B). Intermediate monocytes did to a lesser degree, with a mix of highly adherent macrophage-like cells and occasional cells with dendrites (Fig 4A and 4B). Non-classical monocytes did not, showing differentiation of a few cells to macrophage-like morphology, but massive cell death otherwise (Fig 4A and 4B). After differentiation induction, classical monocytes exhibited a DC immunophenotype, including high expression of HLA-DR, loss of CD14 expression, and acquisition of CD16, CD11c, CD209 (DC-SIGN), CD1a, and CD40; intermediate monocytes maintained expression of CD14 and exhibited lower expression of DC markers than classical monocytes; non-classical monocytes did not shift their surface marker expression to that of a mo-DC (Fig 4C and 4D). In CFSE-MLR mo-DCs derived from classical monocytes were able to stimulate robust allogeneic T cell proliferation (Fig 4E) and IFN-γ production (Fig 4F). Intermediate and non-classical monocytes subjected to the same mo-DC differentiation cocktail, as well as undifferentiated monocytes of each subset, were not able to induce T cell proliferation or IFN-γ production (Fig 4E and 4F). Mo-DC generated in these experiments did not display enhanced phagocytosis of 1μm beads relative to undifferentiated monocytes (Fig 4G), consistent with their DC phenotype.

### Monocytes are an important source of IFN-α but not a precursor of plasmacytoid DC

**Fig 5:**
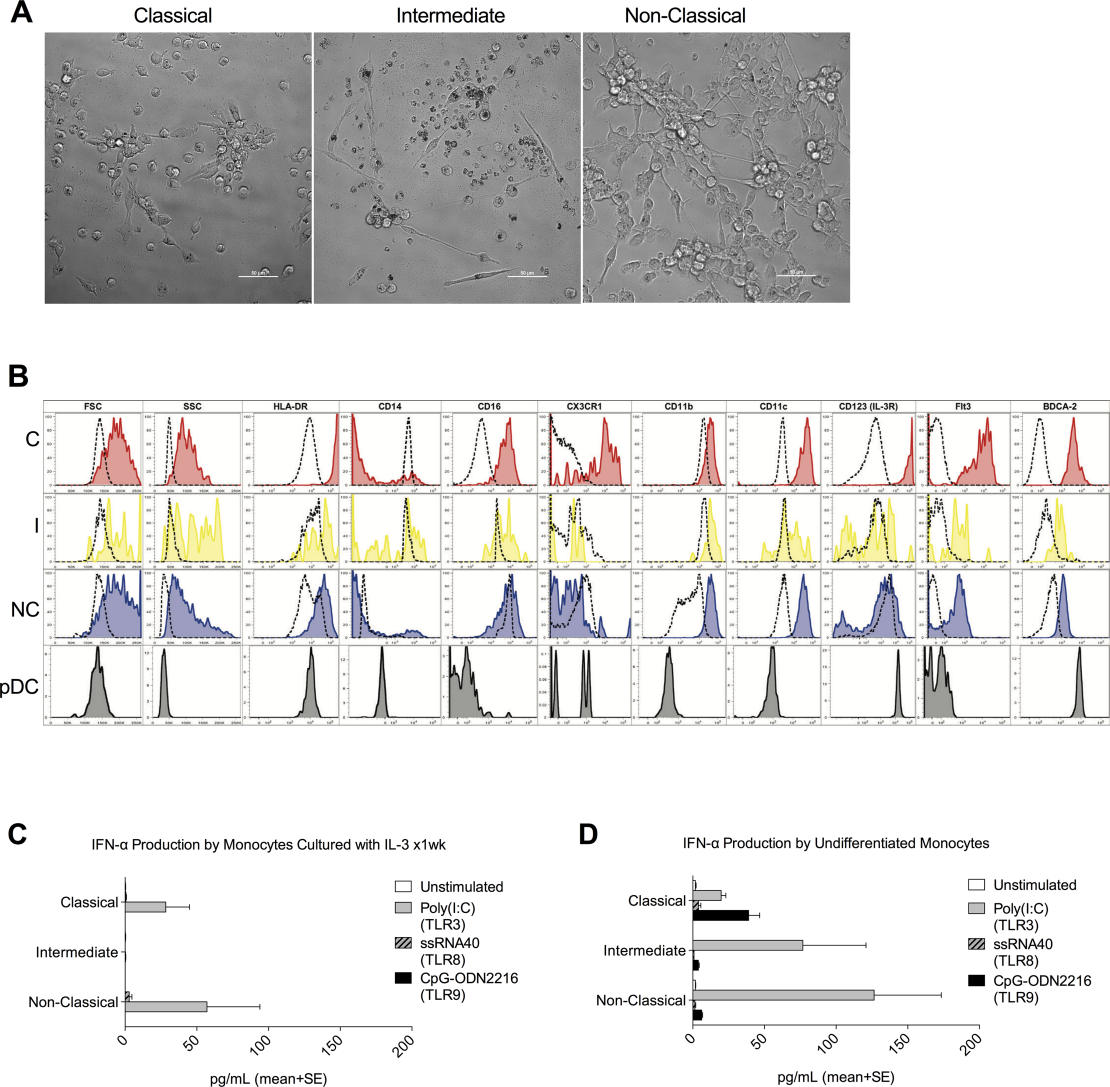
Monocytes do not differentiate to pDCs, but do secrete type I IFN in response to intracellular TLR stimulation. **(A)** Photomicrographs of sorted classical, intermediate, and non-classical monocytes after one week of culture in pDC differentiation medium. All cultures display features of both differentiated DCs and macrophages, with colony formation and reticular networking (bar = 50μm). **(B)** Representative histograms from one of two healthy subjects (C = classical, I = intermediate, NC = non-classical). Classical monocytes (red), intermediate monocytes (yellow), and non-classical monocytes (blue) cultured for seven days in pDC medium are shown overlaid on undifferentiated, uncultured monocytes (dashed). Plots from mature pDCs are shown in the bottom row (grey shaded). **(C)** IFN-α secretion by monocytes cultured in pDC differentiation medium for seven days and stimulated with agonists to intracellular TLR (mean +/- SD, n = 2) **(D)** IFN-α secretion by undifferentiated monocytes in response to stimulation with agonists to intracellular TLR. TLR agonists were added in the presence of lipid transfection reagents in both C and D.

We cultured sorted monocyte subsets with IL-3 and Flt3 ligand to determine their ability to differentiate to pDCs [34]. We did not observe development of pDC morphology (spherical, secretory morphology similar to that of a plasma cell lymphocyte) by any subset (Fig 5A). Classical monocytes differentiated to cells exhibiting DC and macrophage morphology, as did intermediate monocytes, but intermediate monocytes also exhibited high cell death. Non-classical monocytes displayed robust proliferation in pDC induction medium and differentiated to cells with DC and macrophage morphology that formed interconnected reticular networks in the dish. While the classical subset displayed up-regulation of markers associated with human pDCs, such as BDCA-2, IL-3R, and Flt3, other surface markers examined (HLA-DR, CD14, CD16, CD11b, CD11c, CX3CR1) were not in agreement with an immunophenotype of pDCs (Fig 5B). Additionally, we did not observe enhanced IFN-α secretion by any subset in response to intracellular stimulation of TLRs 3, 8, and 9 following induction to pDC differentiation (Fig 5C). Of note, these cells secreted lower levels of IFN-α relative to undifferentiated monocytes (Fig 5D) upon stimulation of TLRs 3, 8, and 9 with Poly(I:C), ssRNA40, and CpG dinucleotides, respectively. Therefore, we did not find convincing evidence that any monocyte subset could be made to differentiate into pDC, but observed significant production of IFN-α by undifferentiated monocytes, especially the non-classical subset, in response to viral TLR ligands.

### All monocyte subsets differentiate to macrophages

**Fig 6:**
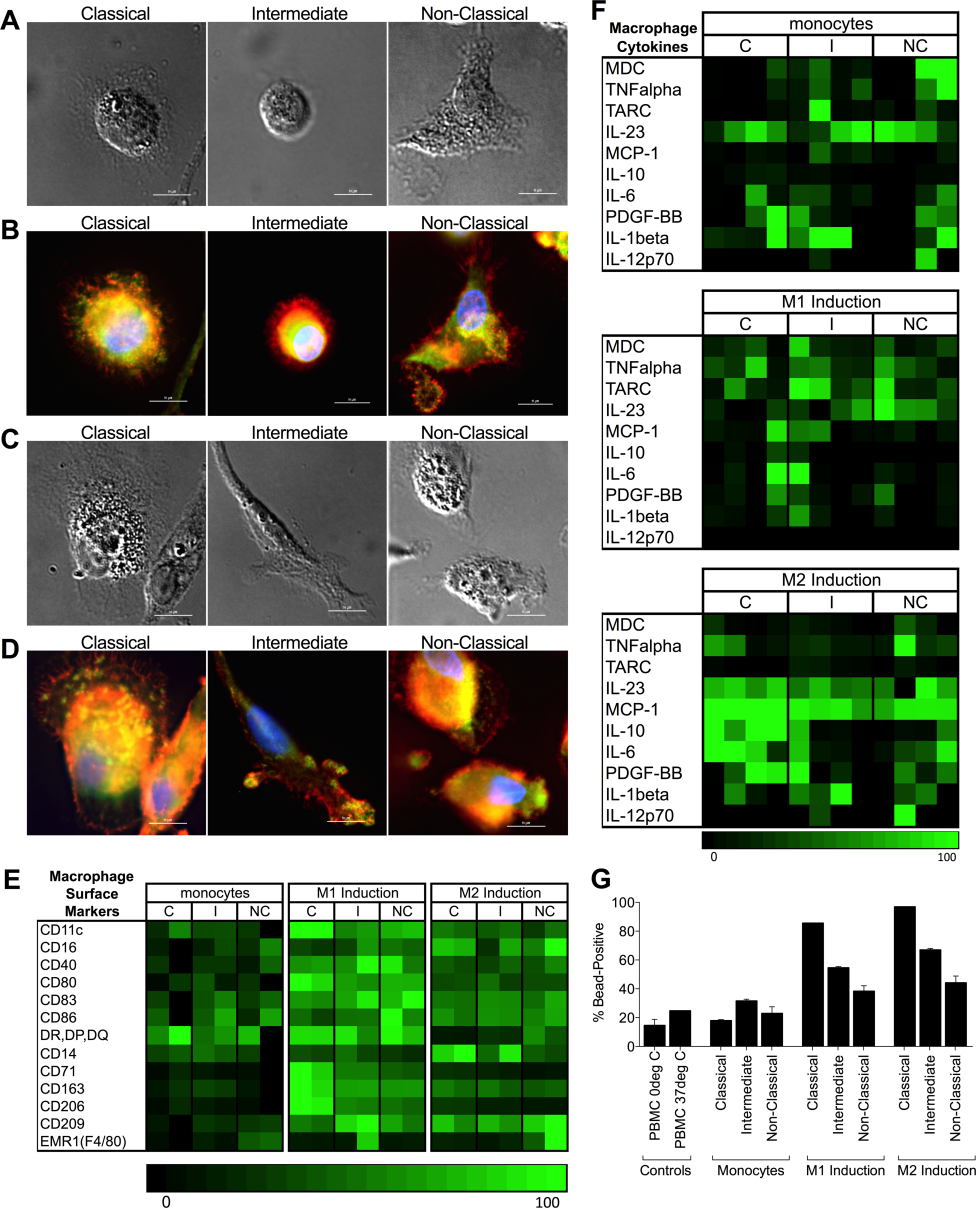
All monocyte subsets differentiate to macrophages with culture in GM-CSF- or M-CSF-containing medium. **(A)** DIC images of macrophages differentiated from sorted classical, intermediate, or non-classical monocytes cultured for five days in medium with low-dose GM-CSF to induce M1 differentiation (bar = 10μm). **(B)** Fluorescent images of the cells in panel A, where actin (orange), vinculin (green), β-tubulin (green) and nucleus (blue) stains show morphological features. Of note, all subsets display rounded but firmly adherent appearance with actin-rich podosomes and extensions indicating attachment to the substrate (bar = 10μm). **(C)** DIC images of macrophages differentiated from sorted classical, intermediate, or non-classical monocytes cultured for five days in medium with M-CSF to induce M2 differentiation (bar = 10μm). **(D)** Fluorescent images of the cells in panel C, where actin (orange), vinculin (green), β-tubulin (green) and nucleus (blue) stains show morphological features. Note the presence of ventral membrane podosome formation in cells from all subsets (bar = 10μm). **(E)** Heatmap showing normalized expression of macrophage-associated surface markers for each subset (C = classical, I = intermediate, NC = non-classical) as undifferentiated, uncultured monocytes (left panel), cultured in M1 medium (center panel), or cultured in M2 medium (right panel). **(F)** Heatmap showing secretion of macrophage-associated cytokines for each subset (C = classical, I = intermediate, NC = non-classical) as undifferentiated, uncultured monocytes (top panel), cultured in M1 medium (middle panel), or cultured in M2 medium (bottom panel). Each box represents the mean of two technical replicates; data is shown separately for each subject tested. **(G)** Phagocytosis by undifferentiated monocytes and monocytes differentiated in either M1 or M2 macrophage-induction medium. Bars represent mean +/- SD of two technical replicates from one subject. Controls include PBMCs incubated with beads at 0°C and 37°C (left bracket).

Investigating the potential of each subset to differentiate to classical (M1) and alternative (M2) macrophages, we observed that all monocyte subsets acquired macrophage morphology, including a rounded but firmly adherent appearance with actin-rich podosomes and extensions indicating attachment to the substrate (Fig 6A–6D). All subsets acquired the expected surface markers in response to incubation with either low dose GM-CSF (to induce M1) or M-CSF (to induce M2) (Fig 6E). In addition, HLA-Class II, CD80, CD71 and CD206 were preferentially up-regulated in all subsets by M1 conditions compared to M2. Similarly, all subsets produced macrophage-associated cytokines in response to both induction media. However, we observed more robust secretion of macrophage-associated cytokines after differentiation with M-CSF (M2) rather than GM-CSF (M1), in particular IL-23 and MCP-1 in all subsets (Fig 6F). We also observed enhanced secretion of IL-10, IL-6, and PDGF-BB from the classical monocyte subset after differentiation with M-CSF. Macrophages differentiated from all subsets demonstrated strong phagocytic activity relative to undifferentiated monocytes or bulk PBMC, with the greatest response observed in the classical monocyte-derived macrophages independent of the induction protocol (GM-CSF vs M-CSF) (Fig 6G).

## Discussion

In this manuscript we sought to expand our understanding of the functional significance of human peripheral blood monocyte subsets because of the important roles that monocytes have in immunity, inflammation, and tissue homeostasis, as well as the diseases that arise when these fundamental processes become dysregulated. Our studies clarified previously reported characteristics of these subsets but also uncovered novel, fundamental features relevant to their function and differentiation potential.

Immunophenotyping of monocyte subsets showed differential expression of surface markers, with higher expression of CD11b and CD62L on classical and intermediate subsets and higher expression of CD11c and CX3CR1 on the non-classical subset. These findings are in agreement with previous work from other groups [21]. However, we noted higher expression of the regulatory molecule SIRPα on non-classical monocytes, which is a novel finding and one that may have implications regarding a regulatory role for non-classical monocytes, especially considering the propensity of this subset to macrophage differentiation and the known regulatory function of SIRPα on tissue-resident macrophages that are important for maintaining immune homeostasis. SIRPα, which is expressed on monocytes and monocyte-derived cells, is an inhibitory receptor that suppresses phagocytosis, DC differentiation and maturation [35] as well as cytokine production based on our current results.

We found that monocyte subset frequencies are tightly regulated in healthy subjects, both over time and across multiple individuals, reinforcing the idea that functional differences between the subsets are of such consequence as to merit strict regulation and maintenance. One of these differences may be in the types of pathogenic stimuli each subset responds to. It has been previously documented that classical and intermediate monocytes respond to bacteria-associated signals, whereas non-classical monocytes are poorly responsive to bacteria-associated signals and more responsive to virus-associated signals [21]. Our findings with regard to cytokine secretion by each human monocyte subset are consistent with this notion.

Our finding in this experimental *in vitro* system suggests that human classical monocytes differentiate to a mo-DC phenotype, while non-classical and intermediate monocyte subsets do not, is in agreement with *in vivo* observations in the mouse that classical monocytes readily follow a DC differentiation program at the expense of macrophage differentiation, while non-classical, patrolling monocytes favor a macrophage differentiation program [17]. Although two earlier studies on human monocytes had suggested that both classical and non-classical subtypes can be made to differentiate into mo-DC, the results may have been biased by the gating strategy and/or method used to sort monocyte subsets, such that a substantial fraction of classical monocytes, which are by far the most numerous, were included in the non-classical subset [33,36]. In contrast, by using a stringent gating strategy whereby non-classical monocytes are identified based on bright CD16 staining (CD16^++^) (Fig 1A), instead of merely CD16^+^ and by utilizing flow-assisted high-speed cell sorting we ensured very high purity of the monocyte subsets subjected to further analysis. Finally, we did not observe significant induction of CD80 or CD83 on mo-DC generated from classical monocytes, suggesting these cells require additional stimulatory cues for maturation [20,37].

We observed that under commonly used macrophage differentiation conditions (GM-CSF or M-CSF), all human monocyte subsets readily undergo differentiation to macrophages, with enhanced phagocytic function. The classical monocyte subset exhibited the most phagocytic function following differentiation. In the absence of added differentiation factors, however, the non-classical subset appeared to have an inherent propensity to differentiate into macrophages. The few non-classical monocytes that adhered to tissue culture plastic in unsupplemented medium displayed signs of macrophage differentiation, such as ventral membrane podosome formation, after just one to two days in culture. This would suggest that the non-classical subset is predisposed to differentiate to macrophages upon egress from circulation, though further study *in vivo* will be required to understand the significance of this observation. Our observation that only classical monocytes can differentiate into either DCs or macrophages points to the remarkable plasticity of this subset in comparison to the other monocyte subtypes and lends further support to the widely circulated but unproven hypothesis that the three monocyte subsets represent discrete stages on a spectrum of differentiation from classical to non-classical, with classical monocytes representing the least differentiated.

We did not distinguish greatly between differentiation to classical (M1) versus alternative (M2) macrophages, given that: (1) tissue macrophages represent a complex collection of cells, many of which do not even originate from circulating monocytes, and they are inadequately represented by this dichotomous terminology; (2) specifically the classification of macrophages within the M2 designation has grown diverse, complex, and context-dependent with the inclusion of studies on cancer, atherosclerosis, and other non-physiologic states in a preponderance of the macrophage literature; and (3) macrophage classifications exist *in vivo* not so much as a terminally differentiated fate, but as an activation status that changes with time and context [2,4,38,39]. Additionally, the lineage markers attributed to each of these phenotypes are not well defined. Rather, we relied upon morphologic features, such as actin-rich podosomes, and phagocytic activity as indicators of a macrophage phenotype and presented the surface marker and cytokine secretion data without strictly assigning them to M1 and M2 subtypes, which overlap. This is not to suggest that M1 and M2 polarizations are not real or of importance, especially when considering the effects monocytes and macrophages have on T helper polarization [40,41], but that additional analysis of monocyte/macrophage polarization is beyond the scope of the current work.

We concluded based on surface marker expression and secretion of IFN-α that no monocyte subset can be made to differentiate to a pDC phenotype using an induction cocktail of IL-3 and Flt3 ligand, which is in agreement with the mouse literature showing that monocytes likely split from a pDC-competent lineage at the level of the macrophage-DC progenitor [42,43], though this is much less clear in humans [44]. Our observation that non-classical monocytes do not differentiate to pDC yet are particularly good producers of type I IFN in response to viral simulants, especially the TLR3 ligand poly(I:C) [45], is consistent with the idea that they serve as an alternate, available source of IFN-α production, complementing the function of pDCs, which respond with IFN-α release to TLRs 7–9 stimulation [46]. IFN-α release in response to TLR3 stimulation proved essential for generation of type-1 polarized (IL-12 producing) DC which are essential for cross-priming, polarization and imprinting migratory properties of anti-tumor functional CD8^+^ T cells [47]. These results may also support a role for non-classical monocytes in the initiation of autoimmune disease through their ability, like pDCs [48], to sense nucleic acids released from human cells [49].

In the presence of IL-3 and Flt3 ligand, all monocyte subsets differentiated to mixed mo-DC/macrophage phenotypes and displayed surface marker expression consistent with mo-DCs, although the intermediate subset was not well sustained in this environment. In particular, non-classical monocytes displayed robust proliferation and formation of reticular networks in response to IL-3 plus Flt3 ligand, while it was not responsive to commonly used protocols for generation of mo-DC *in vitro* (IL-4 + GM-CSF). Flt3 ligand is the primary differentiation cue that drives DC development *in vivo*, but the DCs produced are of lymphoid origin and are generated under steady-state conditions [50,51]. It is generally assumed that Flt3 ligand does not stimulate production of classical (myeloid) DCs or mo-DCs, which *in vivo* arise under inflammatory conditions and are often referred to as inflammatory DCs. Whether non-classical monocytes can give rise to DCs under non-standard conditions *in vitro* or non-inflammatory conditions *in vivo* has not been studied and requires further exploration.

Concepts related to human monocyte subsets that would be helpful to explore in the future include the breadth of the sensing repertoire of each of the subsets and their differentiated progeny; whether and which of these subsets has a regulatory role and is most responsible for generating suppressor myeloid cells; and what the implications are of the different functions and differentiation potential of monocyte subsets to human diseases such as autoimmunity, autoinflammation, transplant rejection, atherosclerosis, and cancer. We believe that the results of our current study lay the ground for informed and accurate analyses of monocyte subsets in these important clinical disorders.

## Supporting information

S1 Table The data of subset percentages for monocyte subsets in healthy human subjects. (XLSX)

S2 Table The data of subset percentages for monocyte subsets in healthy human subjects over time. (XLSX)

S3 Table The data of surface marker expression on healthy human subjects’ monocyte subsets. (XLSX)

S4 Table The data of secreted inflammatory cytokines by each monocyte subset upon stimulation with TLR agonists. (XLSX)

S5 Table The data of surface marker expression on healthy human subjects’ monocyte subsets upon induction to monocyte derived-dendritic cell differentiation. (XLSX)

S6 Table The data of proliferation and IFN positivity of mo-DC differentiated subsets in MLR. (XLSX)

S7 Table The data of fluorescent bead positivity in differentiated monocyte subsets as a measure of phagocytosis. (XLSX)

S8 Table The data of IFNa secretion by monocyte subsets after attempted induction to pDC differentiation. (XLSX)

S9 Table The data of surface marker expression on healthy human subjects’ monocyte subsets upon induction to monocyte derived-dendritic cell differentiation. (XLSX)

S10 Table The data of macrophage-associated cytokine secretion by healthy human subjects’ monocyte subsets upon induction to macrophage differentiation. (XLSX)

## References

[1] Oberbarnscheidt MH, Zeng Q, Li Q, Dai H, Williams AL, Shlomchik WD, et al (2014) Non-self recognition by monocytes initiates allograft rejection. J Clin Invest 124: 3579–3589. doi: 10.1172/JCI74370 24983319 10.1172/JCI74370 PMC4109551

[2] Bigley V, Haniffa M, Doulatov S, Wang XN, Dickinson R, McGovern N, et al (2011) The human syndrome of dendritic cell, monocyte, B and NK lymphoid deficiency. J Exp Med 208: 227–234. doi: 10.1084/jem.20101459 21242295 10.1084/jem.20101459 PMC3039861

[3] Ingersoll MA, Platt AM, Potteaux S, Randolph GJ (2011) Monocyte trafficking in acute and chronic inflammation. Trends Immunol 32: 470–477. doi: 10.1016/j.it.2011.05.001 21664185 10.1016/j.it.2011.05.001 PMC3179572

[4] Hashimoto D, Miller J, Merad M (2011) Dendritic cell and macrophage heterogeneity in vivo. Immunity 35: 323–335. doi: 10.1016/j.immuni.2011.09.007 21943488 10.1016/j.immuni.2011.09.007 PMC4520532

[5] Hill LM, Gavala ML, Lenertz LY, Bertics PJ (2010) Extracellular ATP may contribute to tissue repair by rapidly stimulating purinergic receptor X7-dependent vascular endothelial growth factor release from primary human monocytes. J Immunol 185: 3028–3034. doi: 10.4049/jimmunol.1001298 20668222 10.4049/jimmunol.1001298 PMC3156583

[6] Apostolakis S, Lip GY, Shantsila E (2010) Monocytes in heart failure: relationship to a deteriorating immune overreaction or a desperate attempt for tissue repair? Cardiovasc Res 85: 649–660. doi: 10.1093/cvr/cvp327 19805399 10.1093/cvr/cvp327

[7] Anghelina M, Krishnan P, Moldovan L, Moldovan NI (2006) Monocytes/macrophages cooperate with progenitor cells during neovascularization and tissue repair: conversion of cell columns into fibrovascular bundles. Am J Pathol 168: 529–541. doi: 10.2353/ajpath.2006.050255 16436667 10.2353/ajpath.2006.050255 PMC1606496

[8] Mitchell AJ, Roediger B, Weninger W (2014) Monocyte homeostasis and the plasticity of inflammatory monocytes. Cell Immunol 291: 22–31. doi: 10.1016/j.cellimm.2014.05.010 24962351 10.1016/j.cellimm.2014.05.010

[9] Ziegler-Heitbrock L (2014) Monocyte subsets in man and other species. Cell Immunol 289: 135–139. doi: 10.1016/j.cellimm.2014.03.019 24791698 10.1016/j.cellimm.2014.03.019

[10] Bain CC, Scott CL, Uronen-Hansson H, Gudjonsson S, Jansson O, Grip O, et al (2013) Resident and pro-inflammatory macrophages in the colon represent alternative context-dependent fates of the same Ly6Chi monocyte precursors. Mucosal Immunol 6: 498–510. doi: 10.1038/mi.2012.89 22990622 10.1038/mi.2012.89 PMC3629381

[11] Tolouei Semnani R, Moore V, Bennuru S, McDonald-Fleming R, Ganesan S, Cotton R, et al (2014) Human monocyte subsets at homeostasis and their perturbation in numbers and function in filarial infection. Infect Immun 82: 4438–4446. doi: 10.1128/IAI.01973-14 25114121 10.1128/IAI.01973-14 PMC4249311

[12] Venturini J, Cavalcante RS, Golim Mde A, Marchetti CM, Azevedo PZ, Amorim BC, et al (2014) Phenotypic and functional evaluations of peripheral blood monocytes from chronic-form paracoccidioidomycosis patients before and after treatment. BMC Infect Dis 14: 552 doi: 10.1186/s12879-014-0552-x 25314914 10.1186/s12879-014-0552-x PMC4201701

[13] Jagannathan R, Lavu V, Rao SR (2014) Comparison of the proportion of non-classic (CD14+CD16+) monocytes/macrophages in peripheral blood and gingiva of healthy individuals and patients with chronic periodontitis. J Periodontol 85: 852–858. doi: 10.1902/jop.2013.120658 24001047 10.1902/jop.2013.120658

[14] Novais FO, Nguyen BT, Beiting DP, Carvalho LP, Glennie ND, Passos S, et al (2014) Human classical monocytes control the intracellular stage of Leishmania braziliensis by reactive oxygen species. J Infect Dis 209: 1288–1296. doi: 10.1093/infdis/jiu013 24403561 10.1093/infdis/jiu013 PMC3969552

[15] Zheng J, Liang H, Xu C, Xu Q, Zhang T, Shen T, et al (2014) An unbalanced PD-L1/CD86 ratio in CD14(++)CD16(+) monocytes is correlated with HCV viremia during chronic HCV infection. Cell Mol Immunol 11: 294–304. doi: 10.1038/cmi.2013.70 24531620 10.1038/cmi.2013.70 PMC4085489

[16] Varol C, Yona S, Jung S (2009) Origins and tissue-context-dependent fates of blood monocytes. Immunol Cell Biol 87: 30–38. doi: 10.1038/icb.2008.90 19048016 10.1038/icb.2008.90

[17] Auffray C, Fogg D, Garfa M, Elain G, Join-Lambert O, Kayal S, et al (2007) Monitoring of blood vessels and tissues by a population of monocytes with patrolling behavior. Science 317: 666–670. doi: 10.1126/science.1142883 17673663 10.1126/science.1142883

[18] Carlin LM, Stamatiades EG, Auffray C, Hanna RN, Glover L, Vizcay-Barrena G, et al (2013) Nr4a1-dependent Ly6C(low) monocytes monitor endothelial cells and orchestrate their disposal. Cell 153: 362–375. doi: 10.1016/j.cell.2013.03.010 23582326 10.1016/j.cell.2013.03.010 PMC3898614

[19] Saha P, Geissmann F (2011) Toward a functional characterization of blood monocytes. Immunol Cell Biol 89: 2–4. doi: 10.1038/icb.2010.130 21102535 10.1038/icb.2010.130

[20] Zhou LJ, Tedder TF (1996) CD14+ blood monocytes can differentiate into functionally mature CD83+ dendritic cells. Proc Natl Acad Sci U S A 93: 2588–2592. 8637918 10.1073/pnas.93.6.2588 PMC39841

[21] Cros J, Cagnard N, Woollard K, Patey N, Zhang SY, Senechal B, et al (2010) Human CD14dim monocytes patrol and sense nucleic acids and viruses via TLR7 and TLR8 receptors. Immunity 33: 375–386. doi: 10.1016/j.immuni.2010.08.012 20832340 10.1016/j.immuni.2010.08.012 PMC3063338

[22] Robbins SH, Walzer T, Dembele D, Thibault C, Defays A, Bessou G, et al (2008) Novel insights into the relationships between dendritic cell subsets in human and mouse revealed by genome-wide expression profiling. Genome Biol 9: R17 doi: 10.1186/gb-2008-9-1-r17 18218067 10.1186/gb-2008-9-1-r17 PMC2395256

[23] Ancuta P, Liu KY, Misra V, Wacleche VS, Gosselin A, Zhou X, et al (2009) Transcriptional profiling reveals developmental relationship and distinct biological functions of CD16+ and CD16- monocyte subsets. BMC Genomics 10: 403 doi: 10.1186/1471-2164-10-403 19712453 10.1186/1471-2164-10-403 PMC2741492

[24] Ziegler-Heitbrock L, Ancuta P, Crowe S, Dalod M, Grau V, Hart DN, et al (2010) Nomenclature of monocytes and dendritic cells in blood. Blood 116: e74–80. doi: 10.1182/blood-2010-02-258558 20628149 10.1182/blood-2010-02-258558

[25] Wong KL, Tai JJ, Wong WC, Han H, Sem X, Yeap WH, et al (2011) Gene expression profiling reveals the defining features of the classical, intermediate, and nonclassical human monocyte subsets. Blood 118: e16–31. doi: 10.1182/blood-2010-12-326355 21653326 10.1182/blood-2010-12-326355

[26] Kim WK, Sun Y, Do H, Autissier P, Halpern EF, Piatak M Jr., et al (2010) Monocyte heterogeneity underlying phenotypic changes in monocytes according to SIV disease stage. J Leukoc Biol 87: 557–567. doi: 10.1189/jlb.0209082 19843579 10.1189/jlb.0209082 PMC2858301

[27] Yona S, Jung S (2010) Monocytes: subsets, origins, fates and functions. Curr Opin Hematol 17: 53–59. doi: 10.1097/MOH.0b013e3283324f80 19770654 10.1097/MOH.0b013e3283324f80

[28] Zimmermann HW, Seidler S, Nattermann J, Gassler N, Hellerbrand C, Zernecke A, et al (2010) Functional contribution of elevated circulating and hepatic non-classical CD14CD16 monocytes to inflammation and human liver fibrosis. PLoS One 5: e11049 doi: 10.1371/journal.pone.0011049 20548789 10.1371/journal.pone.0011049 PMC2883575

[29] Yang J, Zhang L, Yu C, Yang XF, Wang H (2014) Monocyte and macrophage differentiation: circulation inflammatory monocyte as biomarker for inflammatory diseases. Biomark Res 2: 1 doi: 10.1186/2050-7771-2-1 24398220 10.1186/2050-7771-2-1 PMC3892095

[30] Pawlowski NA, Kaplan G, Hamill AL, Cohn ZA, Scott WA (1983) Arachidonic acid metabolism by human monocytes. Studies with platelet-depleted cultures. J Exp Med 158: 393–412. 6411852 10.1084/jem.158.2.393 PMC2187343

[31] Larsen E, Celi A, Gilbert GE, Furie BC, Erban JK, Bonfanti R, et al (1989) PADGEM protein: a receptor that mediates the interaction of activated platelets with neutrophils and monocytes. Cell 59: 305–312. 2478294 10.1016/0092-8674(89)90292-4

[32] Passlick B, Flieger D, Ziegler-Heitbrock HW (1989) Identification and characterization of a novel monocyte subpopulation in human peripheral blood. Blood 74: 2527–2534. 2478233

[33] Sallusto F, Lanzavecchia A (1994) Efficient presentation of soluble antigen by cultured human dendritic cells is maintained by granulocyte/macrophage colony-stimulating factor plus interleukin 4 and downregulated by tumor necrosis factor alpha. J Exp Med 179: 1109–1118. 8145033 10.1084/jem.179.4.1109 PMC2191432

[34] Demoulin S, Roncarati P, Delvenne P, Hubert P (2012) Production of large numbers of plasmacytoid dendritic cells with functional activities from CD34(+) hematopoietic progenitor cells: use of interleukin-3. Exp Hematol 40: 268–278. doi: 10.1016/j.exphem.2012.01.002 22245566 10.1016/j.exphem.2012.01.002

[35] Barclay AN, Van den Berg TK (2014) The interaction between signal regulatory protein alpha (SIRPalpha) and CD47: structure, function, and therapeutic target. Annu Rev Immunol 32: 25–50. doi: 10.1146/annurev-immunol-032713-120142 24215318 10.1146/annurev-immunol-032713-120142

[36] Sanchez-Torres C, Garcia-Romo GS, Cornejo-Cortes MA, Rivas-Carvalho A, Sanchez-Schmitz G (2001) CD16+ and CD16- human blood monocyte subsets differentiate in vitro to dendritic cells with different abilities to stimulate CD4+ T cells. Int Immunol 13: 1571–1581. 11717198 10.1093/intimm/13.12.1571

[37] Thomas R, Lipsky PE (1994) Human peripheral blood dendritic cell subsets. Isolation and characterization of precursor and mature antigen-presenting cells. J Immunol 153: 4016–4028. 7523513

[38] Satpathy AT, Wu X, Albring JC, Murphy KM (2012) Re(de)fining the dendritic cell lineage. Nat Immunol 13: 1145–1154. doi: 10.1038/ni.2467 23160217 10.1038/ni.2467 PMC3644874

[39] Mills CD (2012) M1 and M2 Macrophages: Oracles of Health and Disease. Crit Rev Immunol 32: 463–488. 23428224 10.1615/critrevimmunol.v32.i6.10

[40] Hoechst B, Gamrekelashvili J, Manns MP, Greten TF, Korangy F (2011) Plasticity of human Th17 cells and iTregs is orchestrated by different subsets of myeloid cells. Blood 117: 6532–6541. doi: 10.1182/blood-2010-11-317321 21493801 10.1182/blood-2010-11-317321

[41] Krausgruber T, Blazek K, Smallie T, Alzabin S, Lockstone H, Sahgal N, et al (2011) IRF5 promotes inflammatory macrophage polarization and TH1-TH17 responses. Nat Immunol 12: 231–238. doi: 10.1038/ni.1990 21240265 10.1038/ni.1990

[42] Fogg DK, Sibon C, Miled C, Jung S, Aucouturier P, Littman DR, et al (2006) A clonogenic bone marrow progenitor specific for macrophages and dendritic cells. Science 311: 83–87. doi: 10.1126/science.1117729 16322423 10.1126/science.1117729

[43] Onai N, Kurabayashi K, Hosoi-Amaike M, Toyama-Sorimachi N, Matsushima K, Inaba K, et al (2013) A clonogenic progenitor with prominent plasmacytoid dendritic cell developmental potential. Immunity 38: 943–957. doi: 10.1016/j.immuni.2013.04.006 23623382 10.1016/j.immuni.2013.04.006

[44] Collin M, Bigley V, Haniffa M, Hambleton S (2011) Human dendritic cell deficiency: the missing ID? Nat Rev Immunol 11: 575–583. doi: 10.1038/nri3046 21852794 10.1038/nri3046

[45] Matsumoto M, Seya T (2008) TLR3: interferon induction by double-stranded RNA including poly(I:C). Adv Drug Deliv Rev 60: 805–812. doi: 10.1016/j.addr.2007.11.005 18262679 10.1016/j.addr.2007.11.005

[46] Hansmann L, Groeger S, von Wulffen W, Bein G, Hackstein H (2008) Human monocytes represent a competitive source of interferon-alpha in peripheral blood. Clin Immunol 127: 252–264. doi: 10.1016/j.clim.2008.01.014 18342575 10.1016/j.clim.2008.01.014

[47] Muthuswamy R, Wang L, Pitteroff J, Gingrich JR, Kalinski P (2015) Combination of IFNalpha and poly-I:C reprograms bladder cancer microenvironment for enhanced CTL attraction. J Immunother Cancer 3: 6 doi: 10.1186/s40425-015-0050-8 25806105 10.1186/s40425-015-0050-8 PMC4371844

[48] Gilliet M, Cao W, Liu YJ (2008) Plasmacytoid dendritic cells: sensing nucleic acids in viral infection and autoimmune diseases. Nat Rev Immunol 8: 594–606. doi: 10.1038/nri2358 18641647 10.1038/nri2358

[49] Kyogoku C, Smiljanovic B, Grun JR, Biesen R, Schulte-Wrede U, Haupl T, et al (2013) Cell-specific type I IFN signatures in autoimmunity and viral infection: what makes the difference? PLoS One 8: e83776 doi: 10.1371/journal.pone.0083776 24391825 10.1371/journal.pone.0083776 PMC3877094

[50] Naik SH, Sathe P, Park HY, Metcalf D, Proietto AI, Dakic A, et al (2007) Development of plasmacytoid and conventional dendritic cell subtypes from single precursor cells derived in vitro and in vivo. Nat Immunol 8: 1217–1226. doi: 10.1038/ni1522 17922015 10.1038/ni1522

[51] Vremec D, Lieschke GJ, Dunn AR, Robb L, Metcalf D, Shortman K (1997) The influence of granulocyte/macrophage colony-stimulating factor on dendritic cell levels in mouse lymphoid organs. Eur J Immunol 27: 40–44. doi: 10.1002/eji.1830270107 9021996 10.1002/eji.1830270107

